# Electron Transport Chain Is Biochemically Linked to Pilus Assembly Required for Polymicrobial Interactions and Biofilm Formation in the Gram-Positive Actinobacterium *Actinomyces oris*

**DOI:** 10.1128/mBio.00399-17

**Published:** 2017-06-20

**Authors:** Belkys C. Sanchez, Chungyu Chang, Chenggang Wu, Bryan Tran, Hung Ton-That

**Affiliations:** Department of Microbiology & Molecular Genetics, University of Texas Health Science Center, Houston, Texas, USA; KUMC

**Keywords:** actinobacteria, *Actinomyces*, *Mycobacterium*, coaggregation, disulfide bond, oxidoreductases, pilus assembly, protein folding, sortase

## Abstract

The Gram-positive actinobacteria *Actinomyces* spp. are key colonizers in the development of oral biofilms due to the inherent ability of *Actinomyces* to adhere to receptor polysaccharides on the surface of oral streptococci and host cells. This receptor-dependent bacterial interaction, or coaggregation, requires a unique sortase-catalyzed pilus consisting of the pilus shaft FimA and the coaggregation factor CafA forming the pilus tip. While the essential role of the sortase machine SrtC2 in pilus assembly, biofilm formation, and coaggregation has been established, little is known about *trans*-acting factors contributing to these processes. We report here a large-scale Tn*5* transposon screen for mutants defective in *Actinomyces oris* coaggregation with *Streptococcus oralis*. We obtained 33 independent clones, 13 of which completely failed to aggregate with *S. oralis*, and the remainder of which exhibited a range of phenotypes from severely to weakly defective coaggregation. The former had Tn*5* insertions in *fimA*, *cafA*, or *srtC2*, as expected; the latter were mapped to genes coding for uncharacterized proteins and various *nuo* genes encoding the NADH dehydrogenase subunits. Electron microscopy and biochemical analyses of mutants with nonpolar deletions of *nuo* genes and *ubiE*, a menaquinone C-methyltransferase-encoding gene downstream of the *nuo* locus, confirmed the pilus and coaggregation defects. Both *nuoA* and *ubiE* mutants were defective in oxidation of MdbA, the major oxidoreductase required for oxidative folding of pilus proteins. Furthermore, supplementation of the *ubiE* mutant with exogenous menaquinone-4 rescued the cell growth and pilus defects. Altogether, we propose that the *A. oris* electron transport chain is biochemically linked to pilus assembly via oxidative protein folding.

## IMPORTANCE

The Gram-positive actinobacterium *A. oris* expresses adhesive pili, or fimbriae, that are essential to biofilm formation and *Actinomyces* interactions with other bacteria, termed coaggregation. While the critical role of the conserved sortase machine in pilus assembly and the disulfide bond-forming catalyst MdbA in oxidative folding of pilins has been established, little is known about other *trans*-acting factors involved in these processes. Using a Tn*5* transposon screen for mutants defective in coaggregation with *Streptococcus oralis*, we found that genetic disruption of the NADH dehydrogenase and menaquinone biosynthesis detrimentally alters pilus assembly. Further biochemical characterizations determined that menaquinone is important for reactivation of MdbA. This study supports the notion that the electron transport chain is biochemically linked to pilus assembly in *A. oris* via oxidative folding of pilin precursors.

## INTRODUCTION

Found only in Gram-positive bacteria, such as *Actinomyces* spp., *Corynebacterium diphtheriae*, *Bacillus cereus*, streptococci, and enterococci, covalently linked pili, also termed fimbriae, are important virulence determinants (1, 2). These adhesive pilus structures are assembled and linked to bacterial peptidoglycan by conserved transpeptidase enzymes collectively named sortases (3). Initially reported in *C. diphtheriae* (1, 4), sortase enzymes that polymerize pilin subunits into covalently linked pilus polymers are often called pilus-specific sortases, or class C sortases (3, 5). Generally, the housekeeping sortase and nonpolymerizing sortase enzymes anchor pilus polymers to the bacterial cell wall (6–10) via a process similar to cell wall anchoring of surface proteins by the archetype SrtA of *Staphylococcus aureus* (11, 12).

Pili were discovered in many species of *Actinomyces* in the 1970s (13, 14), and early pilus characterizations focused on the fimbriae of *Actinomyces naeslundii* genospecies 2 (15), which was later renamed *Actinomyces oris* (16). In the biofilm-forming actinobacterium *A. oris*, two types of fimbriae have been identified. Type 1 fimbriae—consisting of the pilus shaft FimP and the tip pilin FimQ (17)—mediate bacterial binding to salivary proline-rich proteins on the tooth surface (18). SrtC1, the pilus-specific sortase encoded by the type 1 fimbrial gene locus, specifically catalyzes pilus polymerization of FimP and FimQ (17, 19). Type 2 fimbriae, with SrtC2 as their pilus-specific sortase (19, 20), are made of the pilus shaft FimA and the canonical tip pilin FimB (20). Biofilm formation and *Actinomyces* interactions with oral streptococci in biofilm, termed coaggregation (15), are well-documented phenotypes associated with the type 2 fimbriae since a *fimA* mutant fails to mediate biofilm formation and bacterial coaggregation (20, 21). Nonetheless, it has recently been discovered that a coaggregation factor named CafA hijacks the sortase SrtC2 machine, forming a distinct pilus tip with the pilus shaft FimA independent of FimB (22). It is now clear that CafA is the major coaggregation factor of *A. oris* as deletion of *cafA* results in the same coaggregation defect as *fimA* deletion; additionally, a specific antibody against recombinant CafA or addition of this protein blocks bacterial coaggregation (22). Thus, the coaggregation defect of the *fimA* mutant can be attributed to the loss of CafA pilus assembly on the bacterial cell wall, while the role of FimB in oral colonization and biofilm formation remains unknown.

**FIG 1:**
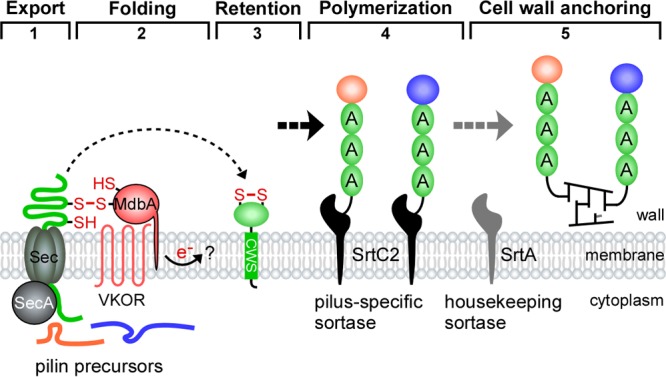
A model of pilus assembly in *A. oris*. Presented is a simplified model of pilus assembly in *A. oris* that is centered on the type 2 fimbriae (see the text for details); FimA, FimB, and CafA are colored green, orange, and blue, respectively. The thiol-disulfide oxidoreductase MdbA catalyzes oxidative folding of nascent protein precursors as they are translocated into the exoplasm by the Sec machinery. Reoxidation of MdbA requires the membrane-bound oxidoreductase VKOR. Folded pilin precursors are polymerized and anchored to the cell wall by the tandem sortase enzymes. It is not known how electrons generated from reoxidation of MdbA/VKOR are transferred (question mark). Dashed arrows denote potential multiple steps (adapted from data in references 22 and 23).

Consistent with the above findings, in a previous small-scale Tn*5* transposon screen intended to identify *A. oris* mutants that fail to aggregate with *Streptococcus oralis*, we found 3 mutants with Tn*5* insertions in *fimA* and *srtC2*; the fourth mutant was mapped to *vkor*, which encodes a bacterial vitamin K epoxide reductase (VKOR) (23). Importantly, an in-frame, nonpolar *vkor* deletion mutant was shown to be severely defective in pilus assembly. Further characterizations established that VKOR is required for reoxidation of the thiol-disulfide oxidoreductase MdbA (23). It is noteworthy that a *Mycobacterium tuberculosis* homolog of *A. oris* VKOR was initially shown to replace DsbB in reoxidation of the disulfide bond forming oxidoreductase DsbA when expressed in *Escherichia coli* (24). Unlike *E. coli dsbA*, *mdbA* is an essential gene, and a conditional *mdbA* deletion mutant failed to assemble adhesive pili (23). It was shown that MdbA and VKOR together form the thiol-disulfide oxidoreductase pair that catalyzes oxidative protein folding in *A. oris* (25). Thus, based on available evidence centered on the type 2 fimbriae, a model of pilus assembly in *A. oris* has been proposed (22, 23) (Fig. 1). Synthesized in the cytoplasm, pilin precursors, such as FimA, FimB, and CafA, are transported across the cytoplasmic membrane by the Sec translocon in an unfolded state. The thiol-disulfide oxidoreductase MdbA catalyzes oxidative folding of nascent pilin precursors before they are embedded into the membrane. Membrane-bound pilin precursors are polymerized by the pilus-specific sortase SrtC2, resulting in formation of type 2 fimbriae with two distinct tip pilins, FimB and CafA. The resulting polymers are presumably anchored by the housekeeping sortase SrtA. While it is clear in this model that the sortase and oxidative folding machines are central elements of pilus assembly in *A. oris*, it is unknown if there are additional factors directly or indirectly involved in the pilus assembly process.

Because *A. oris* coaggregation and pilus assembly are tightly associated, we exploited this property in a high-throughput assay to screen more than 6,200 Tn*5* transposon mutants for clones that are variably defective in coaggregation with *S. oralis* So34, an indicator strain expressing pilus receptor cell-surface polysaccharides (RPS) (20, 21, 26). Using a modified visual coaggregation scoring system, we were able to identify auxiliary factors contributing to pilus assembly that might have been missed in the previously reported small-scale screen, which was solely based on positive or negative coaggregation phenotype scoring (23). By characterizing a subset of these coaggregation-defective mutants, we demonstrate here that NADH dehydrogenase and menaquinone, components of the electron transport chain (ETC), are involved in reoxidation of the major disulfide bond-forming machine MdbA/VKOR in *A. oris*.

## RESULTS

### A Tn*5* transposon screen revealed *A. oris* mutants defective in polymicrobial interactions.

**FIG 2:**
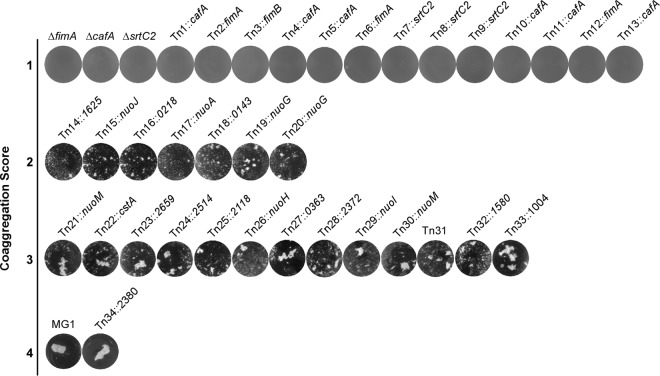
Identification of *A. oris* coaggregation-defective mutants by Tn*5* transposon mutagenesis. Thirty-three *A. oris* coaggregation-defective Tn*5* mutants identified by a cell-based screen were confirmed by a standard coaggregation assay (50). Equal cell numbers of *A. oris* mutants and *S. oralis* So34 were mixed together, and coaggregation was imaged using an AlphaImager. Coaggregation scores indicate the degrees of coaggregation, with the phenotype of the parental MG1 strain scored as 4 and that of the *fimA*, *cafA*, and *srtC2* deletion mutants scored as 1; scores of 2 and 3 represent small and larger clumps of bacterial aggregates. Tn*5* target genes were mapped by TAIL-PCR.

Since pilus assembly and bacterial coaggregation in *A. oris* are coupled (20, 22), we sought to identify *trans*-acting factors involved in pilus assembly by screening a large number of *A. oris* Tn*5* mutants using a previously reported coaggregation assay in a 96-well plate format (23). Following a published protocol (23, 27), a Tn*5* transposon library of roughly 6,240 *A. oris* mutants (>3-fold genome coverage) was constructed and screened. The coaggregation efficiency of the Tn*5* mutants was scored largely based on the visual scoring system reported by Cisar et al. (28), whereby the coaggregation-positive phenotype of the parental strain MG1 was designated 4 and the coaggregation-negative phenotype of type 2 fimbria-less mutants (i.e., Δ*fimA*, Δ*cafA*, and Δ*srtC2*) was set as 1; tiny clumps of aggregates were scored as 2, whereas the larger clumps were scored as 3. We obtained 13 mutants with the coaggregation score (CS) of 1 (CS-1), 7 mutants with the CS of 2 (CS-2), and 13 mutants with CS-3; of note, the Tn*34* mutant exhibiting a coaggregation phenotype similar to that of MG1 was included for comparison (Fig. 2). These coaggregation-defective Tn*5* mutants were then subjected to mapping by thermal asymmetric interlaced PCR (TAIL-PCR) and DNA sequencing as previously described (23).

As expected, 13 coaggregation-negative mutants (i.e., the CS-1 strains) were mapped to genes encoding CafA, FimA, and SrtC2, which are known fimbrial factors essential for *A. oris* coaggregation with oral streptococci (20–22). Intriguingly, 4 out of 7 CS-2 mutants contained Tn*5* insertions in the *nuoA*, *nuoJ*, and *nuoG* genes; the remainder mapped to genes (i.e., ANA_1625, ANA_0218, and ANA_0143) that code for geranylgeranyl reductase (GGR), a putative metal-binding protein, and β-glucosidase, respectively. Finally, the CS-3 mutants were mapped to genes encoding many hypothetical proteins, a carbon starvation protein A (CstA), a two-component system sensor kinase, an ABC-2-type transporter, and the H, I, M subunits of NADH dehydrogenase (i.e., NuoH, NuoI, and NuoM) (Fig. 2; Table 1). Since a large number of the *nuo* genes were repeatedly targeted by the Tn*5* transposon, they were further characterized and reported in this study; the remaining candidates will be investigated in future studies.

**TABLE 1:** Mapping of *A. oris* coaggregation-defective Tn*5* mutants

Tn*5* mutant	Genomic Tn*5* position^a^	Target gene	Predicted function
1	2422225	*cafA*	Coaggregation factor A
2	38189	*fimA*	Type 2 fimbrial shaft pilin
3	36541	*fimB*	Type 2 fimbrial tip pilin
4	2420317	*cafA*	Coaggregation factor A
5	2421799	*cafA*	Coaggregation factor A
6	38174	*fimA*	Type 2 fimbrial shaft pilin
7	39115	*srtC2*	Type 2 pilus-specific sortase
8	39481	*srtC2*	Type 2 pilus-specific sortase
9	39749	*srtC2*	Type 2 pilus-specific sortase
10	2421087	*cafA*	Coaggregation factor A
11	2421136	*cafA*	Coaggregation factor A
12	38721	*fimA*	Type 2 fimbrial tip pilin
13	2420135	*cafA*	Coaggregation factor A
14	1755107	*ana_1625*	Geranylgeranyl reductase
15	1744569	*nuoJ*	NADH dehydrogenase I, subunit J
16	230679	*ana_0218*	Zn^2+^/Mn^2+^ transport system substrate-binding protein
17	1754944	*nuoA*	NADH dehydrogenase I, subunit A
18	162115	*ana_0143*	β-d-Glucoside glucohydrolase
19	1747764	*nuoG*	NADH dehydrogenase I, subunit G
20	1748226	*nuoG*	NADH dehydrogenase I, subunit G
21	1741097	*nuoM*	NADH dehydrogenase, subunit M
22	2348384	*cstA*	Carbon starvation protein A
23	2863369	*ana_2659*	Ni/Fe-hydrogenase III large subunit
24	2711713	*ana_2514*	Histidine kinase (2-component system)
25	2295587	*ana_2118*	Permease component of ABC-type multidrug transport system
26	1746526	*nuoH*	NADH dehydrogenase I, subunit H
27	382186	*ana_0363*	Conserved hypothetical protein
28	2566963	*ana_2372*	Hypothetical protein with Ser/Arg repeats
29	1745086	*nuoI*	NADH dehydrogenase I, subunit I
30	1739922	*nuoM*	NADH dehydrogenase I, subunit M
31	2513949	*ana_2325*; *ana*_*2326*^b^	Ana_2325 (Cys/His-dependent amidohydrolase/peptidase); Ana_2326 (hypothetical protein)
32	1712411	*ana_1580*	Conserved hypothetical protein
33	1080892	*ana_1004*	Hypothetical protein
34	2576712	*ana_2380*	Conserved hypothetical protein

a Shown is the nucleotide position of the Tn*5* transposon mapped in the genome.

b The Tn*5* insertion was found within the intergenic region of *ana_2325* and *ana*_*2326*.

### Genetic disruption of the *A. oris* NADH dehydrogenase (complex I) subunits caused significant defects in CafA-mediated coaggregation and CafA pilus assembly.

**FIG 3:**
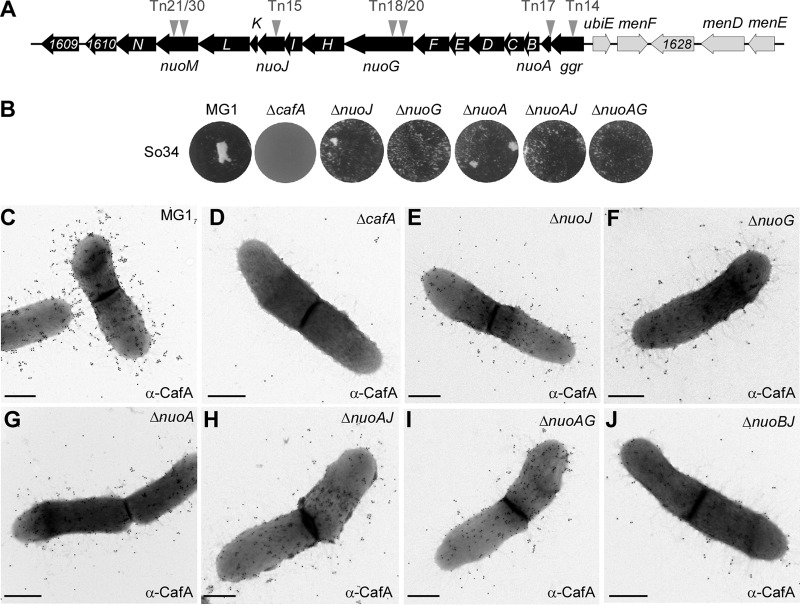
Involvement of *nuo* genes in CafA-mediated coaggregation and pilus assembly. (A) The *nuo* operon and adjacent genes are shown in black, and genes involved in ubiquinone/menaquinone biosynthesis are shown in gray. Arrowheads indicate the locations of the Tn*5* transposon. (B) Coaggregation of *A. oris* strains and *S. oralis* So34 was performed as described in the legend to Fig. 2. (C to J) *A. oris* cells were immobilized in nickel-carbon grids and labeled with anti-CafA antibodies, followed by labeling with anti-rabbit IgG antibodies conjugated to 18-nm gold particles. Samples were stained with 1% uranyl acetate and viewed by a transmission electron microscope. Scale bars indicate 0.5 µm.

The *ggr*, *nuoA*, *nuoG*, *nuoH*, *nuoJ*, and *nuoM* genes revealed from the Tn*5* screen presented above are part of the *nuo* gene locus in *A. oris* MG1 (http://genome.brop.org/), which are predicted to encode the NADH dehydrogenase enzyme—often referred to as the NADH:ubiquinone oxidoreductase or respiratory complex I of the ETC (29) (Fig. 3A). To confirm that the coaggregation defects of the Tn*5*::*nuo* mutants are not due to polar effects, we generated individual mutants and mutants with combinations of in-frame, nonpolar deletions of *nuoJ*, *nuoG*, and *nuoA*. The generated mutants were confirmed for their inability to aggregate with *S. oralis* via CafA-mediated coaggregation using a standard coaggregation assay (22), whereby *A. oris* cells were mixed in equal volume with *S. oralis* So34 cells and coaggregation was determined after a few minutes of mixing. As shown in Fig. 3B, deletion of *nuoJ*, *nuoG*, or *nuoA* or a combination of *nuoA* and *nuoJ* or *nuoA* and *nuoG* caused significant coaggregation defects compared to the wild-type level.

Since CafA pilus assembly is essential for *A. oris* coaggregation (22), we next examined whether the observed coaggregation defects are due to pilus assembly defects of CafA by immunoelectron microscopy (IEM). In this assay, *A. oris* cells were stained with antibodies against CafA (anti-CafA), followed by staining with gold particles conjugated with IgG, and the samples were viewed by transmission electron microscopy (TEM) after staining with 1% uranyl acetate. In the parental strain MG1, CafA signal was found abundantly on the bacterial surface and at the distal end of pili; as expected, the CafA signal was absent in the *cafA* deletion mutant (22). Importantly, CafA labeling was significantly reduced in the *nuoJ*, *nuoG*, *nuoA*, *nuoAJ*, *nuoAG*, and *nuoBJ* mutants (Fig. 3C to J). Since the CafA assembly defects among these mutants were apparently equal, we chose to focus on the *nuoA* mutant for further characterizations (see below) as *nuoA* is typically the first gene in bacterial *nuo* operons (30). To support that the generated *nuoA* mutant is nonpolar, we introduced into this mutant a plasmid constitutively expressing *nuoA*; as shown in Fig. S1 in the supplemental material, ectopic expression of *nuoA* rescued the assembly defects of both type 1 and type 2 pili of the *nuoA* mutant. Of note, the defects in pilus assembly and coaggregation might not be due to the cell growth defect as the growth rate of the *nuoA* mutant was not significantly different from that of the parental strain (see Fig. S3 in the supplemental material). Together, the results confirm the requirement of the NADH dehydrogenase for optimal bacterial coaggregation and CafA pilus assembly.

10.1128/mBio.00399-17.2 FIG S1  Requirement of *nuoA* for pilus assembly. *A. oris* cells of the indicated strains were immobilized on carbon-coated nickel grids and stained with anti-FimA (A to D), anti-CafA (E to H), or anti-type 1 (I to L) antibodies, followed by staining with IgG conjugated to 18-nm gold particles. Samples were stained with 1% uranyl acetate prior to being analyzed by electron microscopy. Scale bars indicate 0.5 µm. Download FIG S1, PDF file, 2.7 MB. Copyright © 2017 Sanchez et al. 2017 Sanchez et al. This content is distributed under the terms of the Creative Commons Attribution 4.0 International license.

### The menaquinone C-methyltransferase UbiE is involved in *A. oris* coaggregation, biofilm formation, and pilus assembly.

In *E. coli*, the NADH dehydrogenase, encoded by 14 *nuo* genes (*nuoA* to -*N*), generates energy by coupling the transfer of electrons from NADH to ubiquinone with the proton translocation across the membrane (29). We observed that upstream of the *A. oris nuo* locus in the MG1 genome are genes encoding part of the ubiquinone/menaquinone biosynthesis pathway, with the C-methyltransferase encoding-gene *ubiE* adjacent to *ggr* (Fig. 3A). In *E. coli*, UbiE catalyzes the carbon methylation reaction in the biosynthesis of ubiquinone and menaquinone, which are essential components of the ETC (31). This information prompted us to examine whether the ubiquinone/menaquinone biosynthesis pathway is also linked to *A. oris* coaggregation and pilus assembly. Since *ubiE* is not an essential gene in *E. coli* (31), we decided to generate an in-frame, nonpolar deletion mutant of *A. oris ubiE*. Using published assays, the generated mutant was examined for its ability to aggregate with *S. oralis*, to mediate biofilm formation, and to produce pili (22).

**FIG 4:**
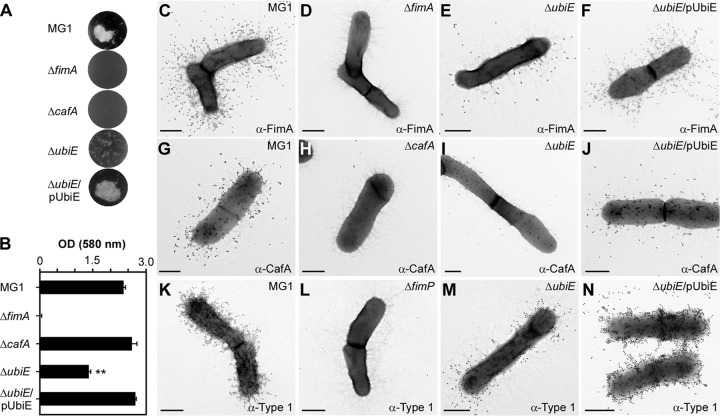
Requirement of *ubiE* for bacterial coaggregation, biofilm formation, and pilus assembly. (A) Coaggregation of *A. oris* strains and *S. oralis* So34 was determined as described in the legend to Fig. 2. (B) Biofilms were obtained by growing the indicated strains in HIB containing 1% sucrose for 48 h. Harvested biofilms were subjected to crystal violet staining and optical density measurement at 580 nm using a microplate reader. The results are shown as representatives of 3 independent experiments performed in triplicate. **, *P* < 0.0025, calculated using a one-way ANOVA (Duncan’s method, nonparametric) with GraphPad Prism. (C to N) *A. oris* cells were immobilized in nickel grids and stained with anti-FimA (C to F), anti-CafA (G to J), or anti-type 1 (K to N) antibodies, followed by staining with IgG conjugated to 18-nm gold particles. Samples were stained with 1% uranyl acetate prior to being analyzed by electron microscopy. Scale bars indicate 0.5 µm.

In the coaggregation assay, the *ubiE* mutant exhibited a severe defect in bacterial coaggregation with *S. oralis* compared to the parental strain, MG1, and this defect was rescued by ectopic expression of UbiE (Fig. 4A). The same set of strains was then examined in biofilm formation assays, in which *A. oris* biofilms were cultivated in heart infusion broth (HIB) supplemented with 1% sucrose, followed by biofilm staining with 1% crystal violet and quantification by the optical density at 580 nm (OD_580_). Consistent with the results described above, deletion of *ubiE* significantly reduced biofilm formation compared to the wild-type level, and expression of *ubiE* in *trans* restored biofilm formation to the wild-type level (Fig. 4B).

To examine if the coaggregation and biofilm defects are associated with pilus assembly, we employed IEM using anti-CafA and antibodies against the type 2 fimbrial shaft FimA (anti-FimA). Compared to the MG1 strain, which produced abundant signals of FimA and CafA, FimA and CafA detection was drastically reduced in the *ubiE* mutant; these defects were rescued in the *ubiE* mutant expressing UbiE from a plasmid (Fig. 4C to J; see Fig. S2 in the supplemental material). Finally, to determine if the role of UbiE also extends to the type 1 fimbriae, we used antibodies against type 1 fimbriae (32) (anti-type 1) in IEM experiments. Consistently, the type 1 fimbrial signal was significantly reduced in the *ubiE* mutant compared to the parental MG1 and UbiE complementing strains (Fig. 4K to N). Altogether, these results suggest that the defects of *ubiE* deletion in bacterial coaggregation and biofilm formation are directly linked to assembly deficiencies of adhesive fimbriae, which are the major factors required for the aforementioned processes in *A. oris* (20, 22).

10.1128/mBio.00399-17.3 FIG S2  Requirement of *nuoA* and *ubiE* for surface expression of CafA. The expression of CafA on the cell surface was determined by whole-cell ELISA using polyclonal anti-CafA antibodies. The absorbance measurements at 450 nm, compared to the *cafA* mutant as background, were determined from three independent experiments performed in triplicate. Error bars represent standard deviations. * and *** indicate *P* < 0.05 and *P* < 0.001, respectively, determined using the unpaired, two-tailed *t* test with GraphPad Prism. Download FIG S2, PDF file, 0.1 MB. Copyright © 2017 Sanchez et al. 2017 Sanchez et al. This content is distributed under the terms of the Creative Commons Attribution 4.0 International license.

10.1128/mBio.00399-17.4 FIG S3  Generation times of the *A. oris* MG1 and Δ*nuoA* mutant strains. Growth of the wild-type (WT) MG1 strain and Δ*nuoA* and Δ*nuoA*/pNuoA mutant strains was measured by optical density (OD_600_). Generation times were calculated as described in Materials and Methods. The results are representative of three independent experiments performed in triplicate. Error bars represent standard deviations. ns, not significant. Download FIG S3, PDF file, 0.1 MB. Copyright © 2017 Sanchez et al. 2017 Sanchez et al. This content is distributed under the terms of the Creative Commons Attribution 4.0 International license.

### Requirement of the menaquinone C-methyltransferase UbiE and NADH dehydrogenase subunit NuoA in reoxidation of the major thiol-disulfide oxidoreductase MdbA.

**FIG 5:**
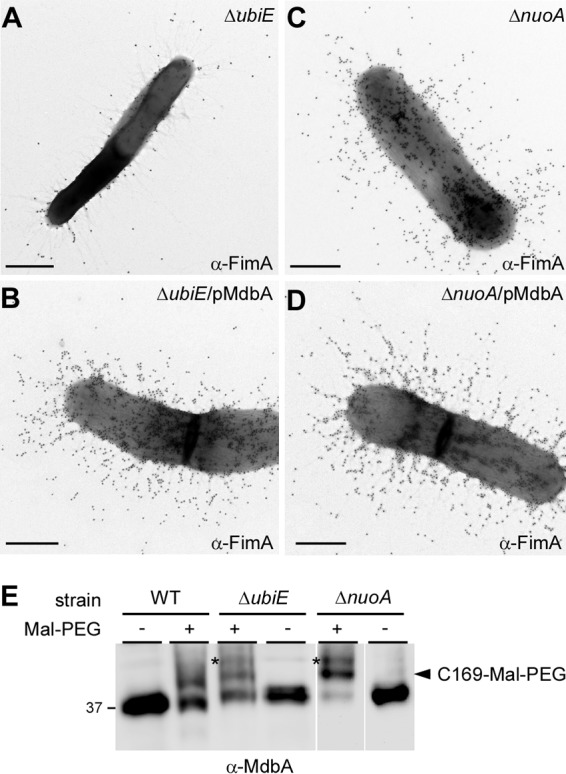
Requirement for NuoA and UbiE in oxidation of the thiol-disulfide oxidoreductase MdbA (A to D). Cells of the indicated *A. oris* strains were subjected to immunogold labeling with anti-FimA as described in the legend to Fig. 4. Scale bars indicate 0.5 µm. (E) Whole-cell lysates of *A. oris* strains were prepared by mechanical disruption and treated (+) or not treated (−) with Mal-PEG. Protein samples were immunoblotted with antibodies against the thiol-disulfide oxidoreductase MdbA (α-MdbA). A reduced form of MdbA is shown by an asterisk, whereas the MdbA species labeled by Mal-PEG at C169 are indicated by a black arrowhead.

As mentioned above, disulfide bond formation is required for correct folding of pilin precursors, and this process is catalyzed by the major thiol-disulfide oxidoreductase MdbA in *A. oris* (23). Given that UbiE and several subunits of the NADH dehydrogenase are associated with pilus assembly, as presented above, we hypothesized that the pilus assembly defects in the *ubiE* and *nuo* mutants might be due to aberrant oxidative folding of pilus proteins. Reactivation of MdbA normally requires the membrane-bound oxidoreductase VKOR, but overexpression of MdbA in a *vkor* mutant can compensate for the loss of VKOR (23). Here, we examined if ectopic expression of MdbA in the absence of UbiE or Nuo subunits would rescue the pilus assembly defects by introducing a recombinant plasmid that constitutively expresses MdbA into the *ubiE* and *nuoA* mutants. The resulting strains, along with the mutants, were examined for their ability to assemble pili by IEM with anti-FimA. Compared to the parental MG1 strain, the *ubiE* mutant produced significantly less FimA (compare Fig. 4C and Fig. 5A), whereas overexpression of MdbA in this mutant resulted in abundant production of FimA pili (Fig. 5B). While the assembly defect of FimA in the *nuoA* mutant was not as severe as that of the *ubiE* mutant, MdbA overexpression in this *nuoA* mutant also increased pilus production (Fig. 5C and D; Fig. S2). These results suggest that MdbA was not sufficiently reoxidized by VKOR in the absence of UbiE or NuoA.

To confirm this, we examined the oxidation status of MdbA by alkylation with methoxypolyethylene glycol-maleimide (Mal-PEG), as previously reported (23, 33). In this assay, protein samples acid trapped by trichloroacetic acid (TCA) precipitation from cell lysates of the MG1, Δ*ubiE*, and Δ*nuoA* strains were treated with 10 mM Mal-PEG or mock treated prior to immunoblotting with antibodies against MdbA (anti-MdbA). As the wild-type MG1 strain contains MdbA with the catalytic CXXC motif C139/142 and a noncatalytic cysteine residue, C169 (23), Mal-PEG treatment caused an upshift in MdbA mobility that was due to Mal-PEG modification of the free sulfhydryl group of C169, as previously reported (23) (Fig. 5E; lanes WT, black arrowhead). Significantly, Mal-PEG treatment of the Δ*ubiE*, and Δ*nuoA* mutants resulted in a higher upshift than that of the MG1 strain under the same condition, consistent with Mal-PEG modification of additional reduced sulfhydryl groups at the active site of MdbA (Fig. 5E, last 4 lanes, asterisks). These results support that the catalytic cysteine residues C139/142 in the CXXC motif are in a reduced form when *ubiE* and *nuoA* are genetically disrupted, very much like the phenotype of MdbA when VKOR is absent (23).

### Exogenous menaquinone rescues the pilus assembly and cell growth defects of the *ubiE* mutant.

**FIG 6:**
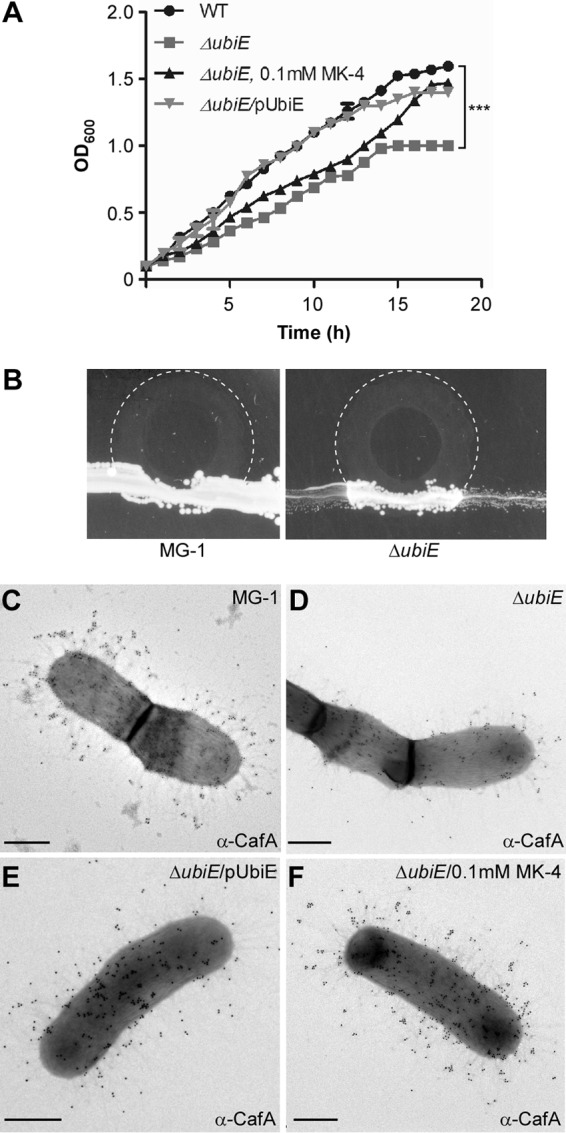
Exogenous menaquinone rescues the growth and pilus assembly defects of the Δ*ubiE* mutant. (A) Growth of the wild-type (WT) strain (solid circles), Δ*ubiE* (gray squares), Δ*ubiE*/pUbiE (gray inverted triangles), and Δ*ubiE* strains in the presence of 0.1 mM menaquinone-4 (MK-4 [triangles]) was measured by optical density (OD_600_). The results are representative of three independent experiments performed in duplicate. ***, *P* ≤ 0.0001, as determined by paired two-tailed *t* test with GraphPad Prism. (B) *A. oris* strains were streaked as a broad band on HIA plates. A 3-μl drop of 50 mM MK-4 was placed on the border of the streaks, and the growth of the strains at 37°C was recorded after 48 h. Areas of MK-4 diffusion are marked with dashed lines. (C to F) *A. oris* cells of the WT and Δ*ubiE*, Δ*ubiE*/pUbiE, and Δ*ubiE* mutants grown in the presence of 0.1 mM MK-4 were subjected to immunogold labeling with anti-CafA as described in the legend to Fig. 4. Scale bars indicate 0.5 µm.

In the majority of Gram-positive bacteria, menaquinone plays a central role in the ETC, functioning as a conduit to receive electrons from electron donors (e.g., NADH dehydrogenase) and transfer them to an electron acceptor (e.g., cytochrome *c* reductase) (34, 35). As UbiE catalyzes the conversion of dimethyl-menaquinone to menaquinone (31, 36), the lack of *ubiE* potentially reduces the quinone pool in the mutant cells, leading to the pleiotropic effects discussed above. To examine this, we monitored the growth rates of *A. oris* strains grown in HIB by optical density (OD_600_) over time. Indeed, the *ubiE* mutant displayed a slow-growth defect compared to the parental strain, and complementation of this strain with the pUbiE plasmid rescued this growth defect (Fig. 6A). Importantly, addition of 0.1 mM menaquinone-4 (MK-4) to the culture medium increased the growth rate to a level comparable to that of the complementing strain after 16 h of growth (Fig. 6A, black and gray triangles).

To further confirm this phenotype, we adapted a streaking assay with menaquinone (37), whereby the parent MG1 strain and its *ubiE* isogenic mutant were streaked as a broad band on the heart infusion agar plate, and a 3-μl drop of 50 mM MK-4 was placed on the border of the streak. Cell growth at 37°C was recorded after 48 h. As shown in Fig. 6B, the MG1 strain exhibited abundant growth inside and outside the MK-4 diffusing zone (dashed lines), whereas the *ubiE* mutant cells inside the MK-4 diffusing zone grew significantly better than those found outside.

Finally, to determine whether menaquinone can also rescue pilus assembly defects, we collected the *ubiE* mutant cells grown in the presence or absence of MK-4 for IEM using anti-CafA. Indeed, the mutant cells grown in the presence of MK-4 produced abundant CafA signal at a level comparable to that of strain MG1 and the complementing strains (Fig. 6C to F). Altogether, the results support that the defects of the Δ*ubiE* mutant in pilus assembly, cell growth, and MdbA reoxidation are mainly due to the reduced level of endogenous menaquinone.

## DISCUSSION

Because pilus assembly and bacterial coaggregation are mutually inclusive in *A. oris*, this inherent property was used in our Tn*5* transposon screen intended to identify factors involved in pilus assembly. This screen revealed numerous mutants that display various defects in *Actinomyces* coaggregation with *S. oralis*. As expected, roughly one-third of the coaggregation-defective mutants were mapped to genes encoding the major pilus shaft FimA, the coaggregation factor CafA, and the pilus sortase machine SrtC2 (Fig. 2). Mapping of the remainder surprisingly uncovered the association of coaggregation with the NADH dehydrogenase (complex I) (Table 1), whose genes are adjacent to the ubiquinone/menaquinone biosynthesis gene locus. By genetically and biochemically characterizing two representative mutants with in-frame, nonpolar mutations Δ*nuoA* and Δ*ubiE*, we have established that the two ETC components, the NADH dehydrogenase and menaquinone, are linked to pilus assembly via reoxidation of the major disulfide bond-forming machine MdbA, which is essential for oxidative folding of pilus proteins in *A. oris* (23, 25).

By electron microscopy, both the Δ*nuoA* and Δ*ubiE* mutants are shown to be defective in pilus assembly, although the latter displays the most striking defects (Fig. 3 and 4). Since overexpression of MdbA in the Δ*ubiE* mutant rescues its pilus defects (Fig. 5), we argued that in the absence of UbiE, the disulfide bond-forming machine MdbA is not fully reoxidized, given that reoxidation of MdbA is required for its activity and oxidative folding of *A. oris* pilus proteins (23). Indeed, by alkylation with Mal-PEG we detected a reduced form of MdbA, although oxidized MdbA was also observed (see Fig. 5E). The latter might be due to inefficient alkylation by Mal-PEG and/or spontaneous oxidation of MdbA during the sampling process. Since menaquinone, 2-methyl-3-polyprenyl-1,4-naphthoquinone, is the main quinone utilized in the ETC of Gram-positive actinobacteria (38), the lack of UbiE certainly reduces the menaquinone pool in the cells, as previously demonstrated in the *E. coli ubiE* mutant (31). In fact, by adding MK-4, a menaquinone with 4 isoprene units, to the Δ*ubiE* cell cultures, we were able to rescue not only the cell growth deficiency but also the pilus assembly defect of the Δ*ubiE* mutant (Fig. 6). The results strongly support that menaquinone is the major quinone source capable of reoxidizing the disulfide bond machine MdbA/VKOR in *A. oris*.

In *E. coli*, DsbA is the primary disulfide bond-forming catalyst, which catalyzes disulfide bond formation of nascent polypeptides transported to the periplasm by the Sec apparatus (39–41). DsbA becomes reduced after catalysis, and reoxidation of DsbA requires DsbB (42, 43). Our results presented here are in line with previous studies in *E. coli* that demonstrate the participation of the respiratory ETC in the reoxidation of the DsbA/DsbB system (44, 45). Kobayashi and colleagues showed that *E. coli* mutants lacking *hemA*—coding for glutamyl-tRNA reductase involved in protoheme and siroheme biosynthesis—or *ubiA* and *menA*, which encode products involved in menaquinone biosynthesis, are defective in reoxidation of DsbA when cells are grown under conditions deficient in protoheme or ubiquinone/menaquinone, respectively (44). By reconstituting the *E. coli* disulfide bond-forming machine DsbA/DsbB with purified components, Bader and colleagues demonstrated that under anaerobic conditions, menaquinone serves as an electron acceptor during DsbA/DsbB reoxidation; under aerobic growth ubiquinone acts as electron acceptor that reoxidizes DsbB, which in turn reoxidizes DsbA (45). It is interesting to note that structural studies of a VKOR homolog of *Synechococcus* sp. reveal the protein is complexed with ubiquinone (46). Thus, it is plausible that a similar mechanism of MdbA/VKOR reoxidation occurs in *A. oris*, with menaquinone presumably acting as an electron acceptor for VKOR during this process; how menaquinone mechanistically reoxidizes MdbA/VKOR remains to be investigated in future studies.

Of note, the effect of the NADH dehydrogenase in MdbA/VKOR reoxidation, which has not been reported before with regard to DsbA/DsbB reoxidation, is somewhat puzzling. In many eukaryotic and prokaryotic systems, complex I (NADH quinone:dehydrogenase) serves as an entry point for electrons to enter the respiratory chain, transferring 2 electrons from NADH to ubiquinone (47). In *E. coli*, the NuoB, NuoD, NuoH, and NuoM subunits form a ubiquinone binding pocket (48). Thus, it is possible that genetic disruption of the Nuo subunits in *A. oris* might disturb electron transfer and/or the quinone pool in the cells. However, the effects seen in the *nuo* deletion mutants in pilus assembly are not as striking as those observed in the *ubiE* mutant, although their defects in coaggregation are obvious (Fig. 3). Thus, it is possible that genetic disruption of the Nuo subunits potentially causes pleiotropic effects. It is likely that many other dehydrogenases, such as the malate and succinate (49), may compensate for the loss of the NADH dehydrogenase.

While the association of the NADH dehydrogenase and menaquinone in pilus assembly is clear, the role of other factors identified from the aforementioned coaggregation-defective screen with Tn*5* mutagenesis is not obvious as many of these factors are hypothetical proteins (Table 1). Except for ANA_0218 and ANA_0143, which are predicted to be a Zn^2+^/Mn^2+^ transport system substrate-binding protein and β-d-glucoside glucohydrolase, respectively, the coaggregation defect of the remainder is not glaring. Given the nature of the two proteins mentioned above, it is more likely they may participate in receptor-related binding than their involvement in pilus assembly. Finally, it is noteworthy that the Tn*5* screen in this study is not saturated, which may explain why a *ubiE* mutant was not detected in this screen. Future Tn*5* screens with multifold coverage designed to directly target pilus assembly will be necessary to reveal any additional novel pilus factors.

## MATERIALS AND METHODS

### Bacterial strains, plasmids, media, and cell growth.

The bacterial strains and plasmids used in this study are listed in Table S1 in the supplemental material. *A. oris* cells were grown in heart infusion broth (HIB) or on heart infusion agar (HIA) plates at 37°C with 5% CO_2_. *S. oralis* cells were grown in HIB containing 1% glucose in a Coy anaerobic chamber. *E. coli* DH5α cells were grown in Luria broth (LB) at 37°C. When required, kanamycin was added to the bacterial cultures at a final concentration of 35 or 50 µg ml^−1^. Reagents were purchased from Sigma unless indicated otherwise.

10.1128/mBio.00399-17.5 TABLE S1  Strains and plasmids used in this study. Download TABLE S1, PDF file, 0.1 MB. Copyright © 2017 Sanchez et al. 2017 Sanchez et al. This content is distributed under the terms of the Creative Commons Attribution 4.0 International license.

### Construction of recombinant plasmids.

The *nuo* promoter and the *nuoA* coding sequence were PCR amplified with *A. oris* MG1 genomic DNA as a template using the primers Pnuo-F-KpnI/PnuoA-R and nuoA-F/ nuoA-R-HindIII (see Table S2 in the supplemental material), respectively, and Phusion DNA polymerase (New England Biolabs). Overlapping PCR was employed to fuse the two sequences accordingly (50). The fused fragment was cloned into the pCW10 vector (50), and the generated plasmid was electroporated into an *A. oris nuoA* deletion strain. Similarly, a *ubiE* complementing plasmid was generated and electroporated into a *ubiE* deletion strain (Table S1).

10.1128/mBio.00399-17.6 TABLE S2  Primers used in this study. Download TABLE S2, PDF file, 0.1 MB. Copyright © 2017 Sanchez et al. 2017 Sanchez et al. This content is distributed under the terms of the Creative Commons Attribution 4.0 International license.

### Gene deletion in *A. oris*.

All *A. oris* nonpolar, in-frame deletion mutants were generated using a *galK* counterselection method described previously (51). In this method, 1-kb fragments up- and downstream of a targeted gene were amplified using appropriate primers (Table S2) and fused using overlapping PCR. The 2-kb fragment was then cloned into the integrative plasmid pCWU2 (Table S1), and the resulting plasmid was electroporated into the *A. oris* strain CW1, which lacks the *galK* gene (27). Cointegrants resulting from a single crossover event were selected on kanamycin-containing HIA plates. Loss of the recombinant plasmid by a second crossover event resulting in wild-type and mutant alleles was selected using medium containing 0.2% 2-deoxygalactose (2-DG). Generated mutants were identified by PCR. For double mutants, such as the Δ*nuoAJ* and Δ*nuoAG* strains, single mutants were used as a starting strain.

### Identification of *A. oris* coaggregation-defective mutants by Tn*5* transposon mutagenesis.

Following a published protocol (51), a library of approximately 6,200 kanamycin-resistant Tn*5* mutants was generated from the parental MG1 strain. This library was used to screen for *A. oris* mutants defective in coaggregation with *S. oralis* using a cell-based screen in 96-well plates as previously reported (23). Coaggregation was ranked from 1 to 4, largely based on the scoring system described by Cisar and colleagues (28), with the coaggregation phenotypes of *A. oris* MG1 and *S. oralis* So34 scored as 4 and the Δ*fimA* mutant and *S. oralis* So34 scored as 1; the coaggregation scores 2 and 3 were designated for small and larger clumps of aggregates, respectively.

Consequently, 33 coaggregation-defective mutants were obtained, and TAIL-PCR was employed to map Tn*5* insertion sites in these mutants, detailed in our published procedures (27, 51). In brief, two sequential PCRs were performed; the first reaction started with a colony of Tn*5* mutants suspended in reaction buffer containing primers Tn5-1 and AD-1 (Table S2) and Apex *Taq* DNA polymerase (Genesee Scientific). The product of this PCR was used for the next one with primers Tn5-2 (Table S2) and AD-1. Finally, the obtained product of this reaction was gel purified and submitted for DNA sequencing using primer Tn5-3 (Table S2). The resulting DNA sequences were subjected to a BLAST search against the MG1 genome (http://www.homd.org/) to identify the Tn*5* insertion sites.

### Immunoelectron microscopy.

Immunogold labeling of *A. oris* cells was performed as previously described, with some modifications (52). Cells were washed once and suspended in 0.1 M NaCl. Seven microliters of bacterial cell suspensions was placed on carbon-coated nickel grids, and then samples were washed with phosphate-buffered saline (PBS) containing 1% bovine serum albumin (BSA), followed by blocking with 0.1% gelatin in PBS–1% BSA. Adhered cells were stained with primary antibodies diluted in PBS–1% BSA (1:100 for anti-FimA, 1:50 for anti-CafA, and 1:1,000 for anti-type 1), followed by staining with IgG antibodies conjugated to 18-nm gold particles (Jackson ImmunoResearch Laboratories, Inc.). Finally, samples were washed with water, stained with 1% uranyl acetate, and analyzed using a JEOL JEM-1400 electron microscope. The results are representative of three independent experiments in which the reported phenotypes were observed at a level of at least 95% in the fields of view.

### Coaggregation assays.

Coaggregation assays were performed as previously described, with some modifications (20, 52). Briefly, stationary-phase cultures of *A. oris* and *S. oralis* strain So34 cells were harvested by centrifugation and suspended in TBS buffer (200 mM Tris-HCl [pH 7.4], 150 mM NaCl, 0.1 mM CaCl_2_). Equivalent cell numbers of *A. oris* and *S. oralis* strains, based on OD_600_, were mixed for a few minutes, and bacterial aggregates were imaged using an AlphaImager.

### Biofilm assays.

*In vitro* biofilm formation assays were performed according to a published protocol (50). Briefly, overnight *A. oris* cell cultures were used to inoculate fresh cultures (1:100 dilution) in 1.5 ml of HIB containing 1% sucrose and kanamycin in 24-well plates. After incubation in a CO_2_ incubator at 37°C for 48 h, the biofilms were gently washed with PBS and dried before staining with 1% crystal violet. After washing the unbound dye, the stained biofilms were subject to ethanol treatment before being quantified by absorbance measurement at 580 nm with a Tecan M1000 microplate reader.

### Cell growth assays.

Cell growth of *A. oris* strains was monitored by a plate assay and optical density (OD_600_) in HIB cultures as previously described (50). For the plate assay, the MG1 strain and the Δ*ubiE* mutant were streaked as a broad band on HIA plates. A 3-ml drop of 50 mM MK-4 in ethanol was placed on the border of the streaks. Cell growth at 37°C was recorded after 2 days. For growth in HIB, overnight cultures were used to inoculate fresh cultures in HIB supplemented with 35 μg ml^−1^ of kanamycin with starting OD_600_ of 0.1. The OD_600_ reading was taken every hour, and the OD values were presented as averages of three independent experiments performed in duplicate. Calculation of generation time was performed using the formulas *k* = (log *N*_*t*_ − log *N*_0_)/0.301 *t* and *g* = 1/*k*, where *N*_0_ and *N*_*t*_ are OD_600_ values at times 0 and *t*, respectively, where *t* is the time elapsed between the *N*_*t*_ and *N*_0_ recordings. *k* corresponds to growth rate, and *g* corresponds to generation time expressed in hours (53). Generation times were determined from at least two independent experiments performed in triplicate. Note that the MG1, Δ*nuoA*, and Δ*ubiE* strains contain an empty vector conferring kanamycin resistance.

### Determination of MdbA redox status by alkylation with Mal-PEG.

Mid-logarithmic cultures of *A. oris* were harvested and suspended in SMM buffer (0.5 M sucrose, 10 mM MgCl_2_, 10 mM maleate [pH 6.8]). Bacterial cell suspensions were treated with mutanolysin at 37°C for 2 h. Protoplasts were collected by centrifugation at 1,500 × *g* for 10 min, suspended in alkylation buffer (100 mM Tris-HCl [pH 6.8], 1% SDS, 1× protease inhibitor) plus 10% TCA, and lysed by mechanical disruption using a microtube homogenizer (BeadBug) with 0.1-mm glass beads (MP Biomedical). The resulting cell lysates were incubated in ice for 30 min prior to acetone wash and air drying. For alkylation, obtained protein samples were suspended in alkylation buffer containing 10 mM Mal-PEG and incubated at 37°C for 1 h, followed by TCA precipitation and acetone wash. All protein samples were suspended in SDS sample buffer, separated by SDS-PAGE, and immunoblotted with anti-MdbA (1:2,000 dilution).

### Statistical analysis.

Statistical analysis in this study was performed using GraphPad Prism 5, with significant differences determined by one-way analysis of variance (ANOVA) (biofilm assays) or the paired two-tailed *t* test (growth curves, generation time, and enzyme-linked immunosorbent assay [ELISA]). The results are presented as the average values from at least two independent experiments performed in triplicate ± standard deviations (SD).

10.1128/mBio.00399-17.1 TEXT S1  Supplemental materials and methods. Download TEXT S1, PDF file, 0.1 MB. Copyright © 2017 Sanchez et al. 2017 Sanchez et al. This content is distributed under the terms of the Creative Commons Attribution 4.0 International license.
