# Supplementary figures and images for: Electron Transport Chain Is Biochemically Linked to Pilus Assembly Required for Polymicrobial Interactions and Biofilm Formation in the Gram-Positive Actinobacterium Actinomyces oris
Source: mBio. 2017 Jun 20;8(3):e00399-17. doi: 10.1128/mBio.00399-17 (PMC5478893; doi:10.1128/mBio.00399-17)

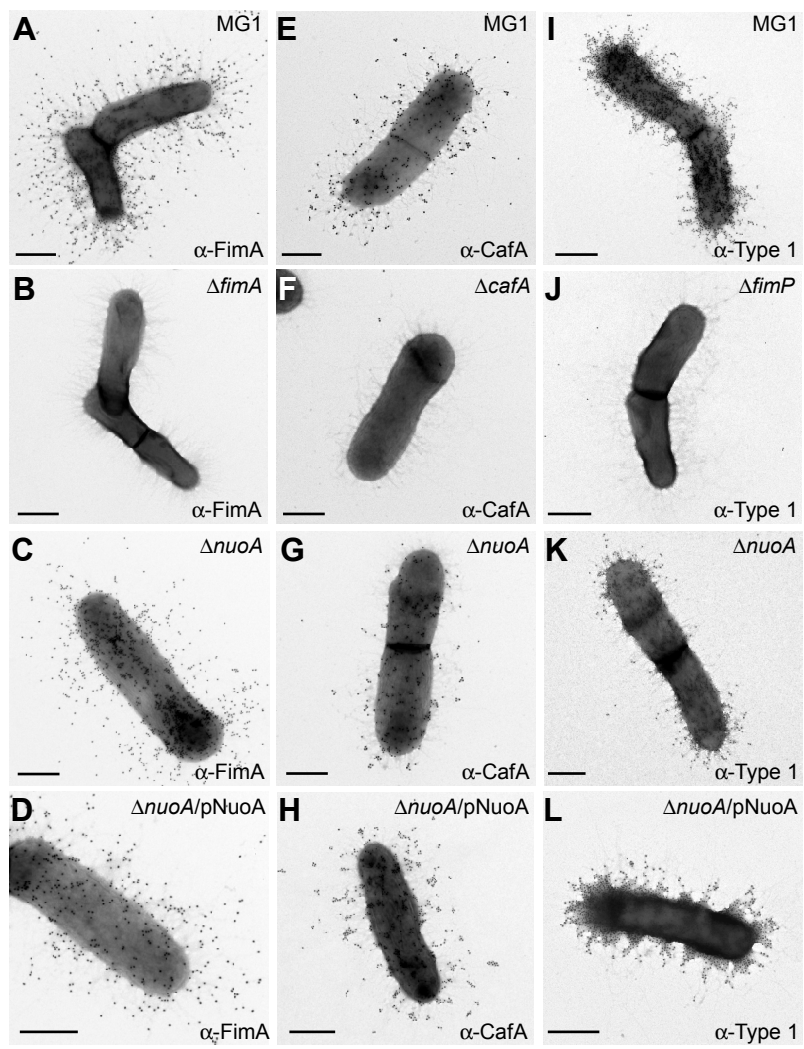

Figure S1: Sanchez et al.

Supplement: FIG S1 [file mbo003173352sf1.pdf]

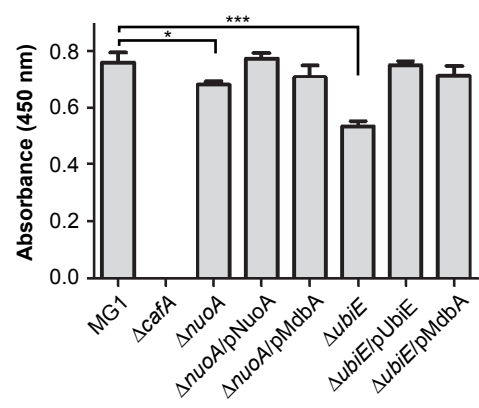

Figure S2: Sanchez et al.

Supplement: FIG S2 [file mbo003173352sf2.pdf]

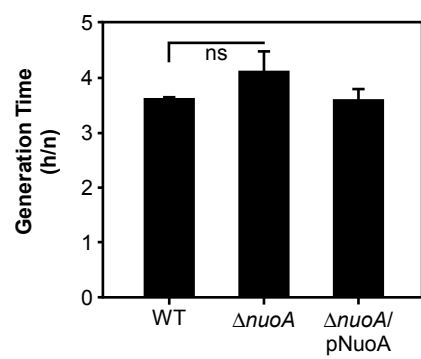

Figure S3: Sanchez et al.

Supplement: FIG S3 [file mbo003173352sf3.pdf]
