# Supplementary material for: Electron Transport Chain Is Biochemically Linked to Pilus Assembly Required for Polymicrobial Interactions and Biofilm Formation in the Gram-Positive Actinobacterium Actinomyces oris
Source: mBio. 2017 Jun 20;8(3):e00399-17. doi: 10.1128/mBio.00399-17 (PMC5478893; doi:10.1128/mBio.00399-17)
Supplement: TABLE S1 [file mbo003173352st1.pdf]

Table S1: Strains and Plasmids used in this study

Strains & Plasmids	Description	Reference
Strain		
A. oris MG1	Parental strain	(2)
A. oris CW1	Δ*galK*; an isogenic derivative of MG1	(2)
A. oris AR4	Δ*fimA*; an isogenic derivative of CW1	(2)
A. oris AR5	Δ*cafA*; an isogenic derivative of CW1	(3)
A. oris JCYC1	Δ*nuoA*; an isogenic derivative of CW1	This study
A. oris JCYC2	Δ*nuoB*; an isogenic derivative of CW1	This study
A. oris BCS3	Δ*nuoG*; an isogenic derivative of CW1	This study
A. oris JCYC3	Δ*nuoJ*; an isogenic derivative of CW1	This study
A. oris JCYC5	Δ*nuoAG*; an isogenic derivative of CW1	This study
A. oris JCYC6	Δ*nuoAJ*; an isogenic derivative of CW1	This study
A. oris BCS2	Δ*nuoBJ*; an isogenic derivative of CW1	This study
A. oris BCS10	Δ*ubiE*; an isogenic derivative of CW1	This study
A. oris BCS4	JCYC1 containing pNuoA	This study
A. oris BCS13	JCYC1 containing pMdbA	This study
A. oris BCS11	BCS10 containing pUbiE	This study
A. oris BCS14	BCS10 containing pMdbA	This study
S. oralis So34	RPS-positive	(4)
Plasmids		
pCWU2	Integrative plasmid expressing the galactokinase *galK* gene under the control of the *rpsJ* promoter	(2)
pCWU2-NuoA	pCWU2 allelic replacement of *nuoA*	This study
pCWU2-NuoB	pCWU2 allelic replacement of *nuoB*	This study
pCWU2-NuoG	pCWU2 allelic replacement of *nuoG*	This study
pCWU2-NuoJ	pCWU2 allelic replacement of *nuoJ*	This study
pCWU2-UbiE	pCWU2 allelic replacement of *ubiE*	This study
pCWU10	*E. coli*/*A. oris* shuttle vector; kanamycin resistant	(5)
pNuoA	pCWU10 expressing *A. oris* wild-type *nuoA*	This study
pUbiE	pCWU10 expressing *A. oris* wild-type *ubiE*	This study
pJRD215	*E. coli*/*Actinomyces* shuttle vector; kanamycin and streptomycin resistant	(6)
pMdbA	pJRD215 expressing *A. oris* wild-type *mdbA*	(7)
