# Supplementary material for: Electron Transport Chain Is Biochemically Linked to Pilus Assembly Required for Polymicrobial Interactions and Biofilm Formation in the Gram-Positive Actinobacterium Actinomyces oris
Source: mBio. 2017 Jun 20;8(3):e00399-17. doi: 10.1128/mBio.00399-17 (PMC5478893; doi:10.1128/mBio.00399-17)
Supplement: TABLE S2 [file mbo003173352st2.pdf]

Table S2: Primers used in this study

Primer	Sequence[(a)]	Application
Ana1624-A-KpnI	AAAAAGGTACCGTTGAGGAGCATCTCGGGGC	*nuoA* deletion
Ana1624-B	CCCATCCACTAAACTTAAACAGACTGAAGCCGCTCTGACCG	*nuoA* deletion
Ana1624-C	TGTTTAAGTTTAGTGGATGGGGGGGTTCATGCTTCCGGCAC	*nuoA* deletion
Ana1624-D-HindIII	AAAAAAGCTTCCCAGGCCACCGCCATCGAC	*nuoA* deletion
Ana1623-A- KpnI	AAAAAGGTACCCGTGGGTCAGGCTCATGAGCTGG	*nuoB* deletion
Ana1623-B	CCCATCCACTAAACTTAAACAGTCGAGGCCGCCGCGCTGTC	*nuoB* deletion
Ana1623-C	TGTTTAAGTTTAGTGGATGGGGGCGTTGTGCTTCTTCATGCTCTTCAC	*nuoB* deletion
Ana1623-D-HindIII	AAAAAAGCTTGCGCAAGCGCCTCATGAAGC	*nuoB* deletion
Ana1615-A-KpnI	AAAAAGGTACCGTTCCTCGGCGGGCAGGCCC	*nuoJ* deletion
Ana1615-B	CCCATCCACTAAACTTAAACAGTCTCCCCATCACCGTCTACCTC	*nuoJ* deletion
Ana1615-C	TGTTTAAGTTTAGTGGATGGGCAGCAGGGTGCTCATCGCAG	*nuoJ* deletion
Ana1615-D-HindIII	AAAAAAGCTTTGATGCCCCGCTACCATGGTC	*nuoJ* deletion
Ana1618-A-KpnI	AAAAAGGTACCAGTGCGTGGACCAGCCGCCCAGG	*nuoG* deletion
Ana1618-B	CCCATCCACTAAACTTAAACACTCCCAGGTCACCGTGACCCATGCAGCGG	*nuoG* deletion
Ana1618-C	TGTTTAAGTTTAGTGGATGGGCCGCGGGTGCGGAGCTCTTGGTGG	*nuoG* deletion
Ana1618-D-HindIII	AAAAAAGCTTCGGCGGCATCCGTCCCGGCC	*nuoG* deletion
Ana1626- A-HindIII	CGCAAGCTTCGGGCCGAGACACCTCCG	*ubiE* deletion
Ana1626-B	GGGCACGGGCCGCGTGGCAATCCCGGCCGCGGTCAC	*ubiE* deletion
Ana1626-C	GTGACCGCGGCCGGGATTGCCACGCGGCCCGTGCCC	*ubiE* deletion
Ana1626-D-HindIII	CGCAAGCTTCGTACCGGCCAGCACCC	*ubiE* deletion
pUbiE-F-HindIII	CGCAAGCTTGCCAGCCGGTGGTGTCCA	*ubiE* complementation
pUbiE-R-EcoRI	CGCGAATTCAGGTGATCGCGCCCAGCG	*ubiE* complementation
Pnuo-F-KpnI	AAAAAGGTACCTAGGACACAGGTCCCGTCCGAC	*nuoA* complementation
PnuoA-R	CCATCCTTGTCAGGAGCTGGTCGGGCTTCCCTCCTCGG	*nuoA* complementation
nuoA-F	CCAGCTCCTGACAAGGATGGTGC	*nuoA* complementation

nuoA-R-HindIII	AAAAAAAGCTTGGCTTCAGTCCCAGTCCAGTCCG	*nuoA* complementation
Tn5-1	CGAACTGTTCGCCAGGCTCAAG	TAIL-PCR
Tn5-2	CTGACCGCTTCCTCGTGCTTTA	TAIL-PCR
Tn5-3	GCCTTCTTGACGAGTTCTTCTGAGCG	Sequencing
AD-1	SWGAXAWGAA[b]	TAIL-PCR

[a]Underlined are restriction site sequences.

[b]X(A/G/C/T), S(G/C), and W(A/T)
