# Supplementary material for: Electron Transport Chain Is Biochemically Linked to Pilus Assembly Required for Polymicrobial Interactions and Biofilm Formation in the Gram-Positive Actinobacterium Actinomyces oris
Source: mBio. 2017 Jun 20;8(3):e00399-17. doi: 10.1128/mBio.00399-17 (PMC5478893; doi:10.1128/mBio.00399-17)
Supplement: TEXT S1 [file mbo003173352s1.pdf]

Electron transport chain is biochemically linked to pilus assembly required for polymicrobial interactions and biofilm formation in the Gram-positive actinobacterium *Actinomyces oris*

Belkys C Sanchez,[1*] Chungyu Chang,[1*] Chenggang Wu,[1] Bryan Tran,[1] and Hung Ton-That[1†]

[1]*Department of Microbiology & Molecular Genetics, University of Texas Health Science Center, Houston, TX, USA.*

[†] Address correspondence to Hung Ton-That, ton-that.hung@uth.tmc.edu

* BCS and CC contributed equally to this work.

Running Head: *ETC linked to pilus assembly via oxidative folding*

SUPPLEMENTAL MATERIAL

Supplemental Materials and Methods

Whole cell ELISA

This experiment was performed according to a published protocol with some modifications (1). Overnight cultures of *A. oris* strains were harvested and suspended in carbonate-bicarbonate buffer (15mM sodium carbonate, 35mM sodium bicarbonate, pH 9.6). Bacterial cells of different strains in equal numbers were used to coat high binding 96-well polystyrene plates, which were incubated for 1 h at 37^{0}C. Plates were washed with PBS containing 0.05% Tween 20 (PBS-T), and blocked with 2% BSA in PBS-T for 1 h at 25^{0}C. After removing blocking solution, plates were incubated for 2 hours at 25^{0}C, with α-CafA (1:5,000) diluted in 1% BSA in PBS-T. Next, plates were washed with PBS-T, and incubated for 1 h at 25^{0}C with secondary antibody conjugated to HRP (1:20,000) diluted in 1% BSA in PBS-T, followed by the 3,3',5,5' tetramethylbenzidine (TMB). The reaction was quenched by addition of 1M H_2SO_4, and the absorbance at 450 nm was measured using a plate reader (Tecan Infinite M1000).

Supplemental Figure Legends

Figure S1: Requirement of *nuoA* for pilus assembly. *A. oris* cells of indicated strains were immobilized on carbon-coated n nickel grids and stained with α-FimA (**A**-**D**), α-CafA (**E**-**H**), or α-Type1 antibodies (**I**-**L**), followed by staining with IgG conjugated to 18-nm gold particles. Samples were stained with 1% uranyl acetate prior to be analyzed by electron microscopy. Scale bars indicate 0.5 μm.

Figure S2: Requirement of *nuoA* and *ubiE* for surface expression of CafA. (A) The expression of CafA on the cell surface was determined by whole cell ELISA using polyclonal α-CafA antibodies. The absorbance measurements at 450nm, as compared to *cafA* mutant as background, were determined from three independent experiments performed in triplicate. Error bars represent standard deviations. *, *** indicate $P<0.05$, $P<0.001$, respectively, which were determined using the unpaired, two-tailed *t*-test with GraphPad Prism.

Figure S3: Generation times of the A. oris MG1 and Δ*nuoA* mutant strains. (A) Growth of the wild-type MG1, Δ*nuoA* and Δ*nuoA*/pNuoA strains was measured by optical density (OD_{600}). Generation times were calculated as described in materials and methods. The results are representative of three independent experiments performed in triplicate. Error bars represent standard deviations, with ns for not significant.

References

1. **Broadway MM, Rogers EA, Chang C, Huang IH, Dwivedi P, Yildirim S, Schmitt MP, Das A, Ton-That H.** 2013. Pilus Gene Pool Variation and the Virulence of *Corynebacterium diphtheriae* Clinical Isolates during Infection of a Nematode. J Bacteriol **195:**3774-3783.
2. **Mishra A, Wu C, Yang J, Cisar JO, Das A, Ton-That H.** 2010. The *Actinomyces oris* type 2 fimbrial shaft FimA mediates co-aggregation with oral streptococci, adherence to red blood cells and biofilm development. Mol Microbiol **77** 841–854.
3. **Reardon-Robinson ME, Wu C, Mishra A, Chang C, Bier N, Das A, Ton-That H.** 2014. Pilus hijacking by a bacterial coaggregation factor critical for oral biofilm development. Proc Natl Acad Sci U S A **111:**3835-3840.
4. **Wu C, Mishra A, Yang J, Cisar JO, Das A, Ton-That H.** 2011. Dual function of a tip fimbrillin of *Actinomyces* in fimbrial assembly and receptor binding. J Bacteriol **193:**3197-3206.
5. **Siegel SD, Wu C, Ton-That H.** 2016. A Type I Signal Peptidase Is Required for Pilus Assembly in the Gram-Positive, Biofilm-Forming Bacterium *Actinomyces oris*. J Bacteriol **198:**2064-2073.
6. **Mishra A, Das A, Cisar JO, Ton-That H.** 2007. Sortase-Catalyzed Assembly of Distinct Heteromeric Fimbriae in *Actinomyces naeslundii*. J Bacteriol **189:**3156-3165.
7. **Reardon-Robinson ME, Osipiuk J, Chang C, Wu C, Jooya N, Joachimiak A, Das A, Ton-That H.** 2015. A Disulfide Bond-forming Machine Is Linked to the Sortase-mediated Pilus Assembly Pathway in the Gram-positive Bacterium *Actinomyces oris*. J Biol Chem **290:**21393-21405.
